# Traveller's Tale from You Nice States

**Published:** 1990-03

**Authors:** A. E. Adam


### TRAVELLER'S TALE FROM YOU NICE STATES

The chief obstacle to an understanding of medicine in the USA is the foreign language they speak there. It is called *Murrican*. The following conversation in Anglo-Murrican took place ten years ago and there is no reason to believe the terminology is any less confusing today than it was then.

"Tell me about this AFIP* place where you were working," said the weekend guest from Croydon over the second Martini.

"The Instoot?" said the pathologist slipping imperceptibly into mid-Atlantic drawl. Two years in the federal capital at the taxpayers' expense had left their mark. "Would you believe that the world's largest, most specialized and most expensive histopathology lab is an *Army* Unit?"

"Really?" murmured the guest, himself ex-RN. "Marvellous how those chaps always fiddle things."

"Well tri-Service really, but run by the US Army. Over 500 staff, nearly 100 pathologists. All in a huge concrete bomb shelter, five stories above ground and three below. A warren of tissoo labs, EM rooms, IBMs and Coke machines. Windowless, nuclear bomb proof . . . "

"I had no idea your work was so important. What did you all do there?"

"Give second opinions on glass slides. And seconds and thirds."

"Never firsts?"

"Nope." The evening and the accent were drawing on. "Yuh see, Ay Eff Eye Pee's mission is tuh give free consultation and dire noses to any—but *any*—pathologist world wide. And tuh advise Uncle Sam on pathology matters."

"Just on this histo—thing?"

"They did some heme and chem path too, but small potatoes."

"It sounds very—recondite."

"Right on! Lookit, horses are commoner than zebras, but at the Instoot horses don't even make the starting line. It's zebras all the way. 50,000 zebras a year. Know how they wrangle them? By sooperspecialization!"

"I don't quite . . . "

* The Armed Forces Institute of Pathology, Washington DC.

"They have a different department for every anatomic part. There's a duh-vision of the Gentle Tract, which includes the Praw State of course, and one for Soft Tissoo two mores. There's a big Duh-vision of Pullman hairy and Meaty ass an' all . . . "

"I *beg* your pardon!"

" . . . for lungs and plurr. Then there's a Very neary in Duh-vision for Zoe knows his, and a Duh-vision of Raid yer logic path to curry late histology with wrenchin ya large cool parents. See Vee Ess is small time-mostly carny REs. I worked in Guy knee . . . "

"Please stop," said the Croydonian weakly, (himself not even a sign tist). "It's all too confusing. Tell me about the people. Are they all military pathologists?"

"No, there's a mix with civilyuns. Incidentally, Murrican pathologists call themselves *fuzz sessions* pry merrily. Even if they are friendsick and only examine stiffs."

"But why?"

"Yuh got three kinds of ducter in the USA. You got the Em Dee – he's a prupper ducter, and likes to be called a fuzz session. Then you got the very neary in and the dennis. They all wear white coats so you can't tell 'em apart."

"Could I have a soft drink please?" said the guest. "I'm getting a headache."

The pathologist broached a Coke and considered. "Yessir, sooperspecialization is the thing. Part of the Murrican wave life. Gin you wine as blueberry pie."

"Do change the subject dear", said his long suffering wife, looking up from sewing Union Jacks onto a table cloth for the church bazaar. "You don't have the faintest idea of what it *feels* like to be an American. How could you, when your childhood never included the Gettysburg address, Little League baseball, milk shakes and drive-ins, heavy dates at 12 and your first Chevvy at 15?"

"Aw, c'mon . . . "

"Did you ever lay your soul on the line for a High School ball game, an Alpha Pi Omega election, the Student Most likely to Succeed award? Did you agonize over Nixon, march against pollution, flagellate over Viet Nam, support an ethnic sensitivity group? These people have suffered in their birth pangs. That sets them apart from the average Brit."

All of which, lazen gemmen, is true. The You Nice States is a long way off from the West Country.

A. E. Adam

